# Freeze-Drying Blue Crab Roe, Sea Urchin, and Beluga Caviar: Impact on Nutritional, Biochemical, and Sensory Properties

**DOI:** 10.3390/md24040135

**Published:** 2026-04-12

**Authors:** Antonia Angou, Spyros Didos, Konstantina Tsotsouli, Ioannis S. Boziaris, Anagnostis Argiriou

**Affiliations:** 1 Institute of Applied Biosciences, Centre for Research and Technology Hellas, 57001 Thessaloniki, Greece; aggouantonia@gmail.com (A.A.); sdidos@certh.gr (S.D.); ktsotsouli@certh.gr (K.T.); 2 Department of Food Science and Nutrition, University of the Aegean, 81400 Lemnos, Greece; 3 Department of Ichthyology and Aquatic Environment, School of Agricultural Sciences, University of Thessaly, 38446 Volos, Greece; boziaris@uth.gr

**Keywords:** freeze-drying, blue crab roe, sea urchin, beluga caviar, flavoring agent, volatile compounds, sensory analysis

## Abstract

The growing demand for clean-label food ingredients drives interest in novel marine flavorings. This study evaluated the physicochemical, antioxidant, volatile (GC-MS), and sensory profiles of freeze-dried powders from blue crab roe (*Callinectes sapidus*), sea urchin roe (*Paracentrotus lividus*), and beluga caviar (*Huso huso*) to assess their culinary potential. Results revealed that sensory quality is governed by the synergy between a matrix’s lipid composition and endogenous antioxidant capacity. Sea urchin powder, possessing a low polyunsaturated fatty acid (PUFA) profile and high carotenoid content, exhibited exceptional oxidative stability, yielding a concentrated marine aldehyde signature and top consumer scores. Blue crab roe demonstrated a robust PUFA matrix buffered by high phenolic content, facilitating controlled lipid peroxidation into desirable savory volatiles (ketones and aldehydes). Conversely, the high-fat, monounsaturated-dominant beluga caviar lacked sufficient antioxidants, leading to lipid degradation, oxidized hydrocarbons, earthy off-flavors, and poor texture. Both crab and caviar powders exhibited favorable Atherosclerosis and Thrombogenicity indices. Ultimately, balancing lipid composition and endogenous antioxidants is crucial for flavor stability, highlighting the commercial and environmental potential of transforming underutilized or invasive species like blue crab into stable, nutrient-dense marine flavoring agents.

## 1. Introduction

The global demand for premium seafood ingredients has grown steadily in recent years, driven by increasing consumer interest in high-value marine products, gourmet cuisine, and natural flavor enhancers [1,2,3]. Blue crab roe, sea urchin, and beluga caviar represent some of the most highly prized seafood commodities, each characterized by unique biochemical profiles, rich umami qualities, and distinctive sensory attributes. These ingredients owe their culinary and economic value to complex matrices of proteins, lipids, amino acids, nucleotides, pigments, and volatile compounds [4,5,6,7,8,9]. However, their extreme perishability, high moisture content, and vulnerability to enzymatic degradation limit their shelf life, restrict their geographic distribution, and complicate their utilization in processed foods [5,9,10].

Freeze-drying has emerged as one of the most effective preservation techniques for high-end and thermally sensitive foods [11,12,13,14]. By removing water under low-temperature and vacuum conditions, freeze-drying minimizes thermal damage, preserves structural and biochemical integrity, and extends product stability while retaining much of the original nutritional value [12,15,16,17,18]. Despite its widespread use for fruits, vegetables, and some marine products, research on the freeze-drying behavior of luxury roe-based ingredients remains limited. Understanding how freeze-drying affects the nutritional composition, lipid oxidation pathways, pigment stability, and volatile flavor profiles of blue crab roe, sea urchin, and beluga caviar is critical for determining their suitability as ingredients in next-generation seafood-derived seasonings and flavoring agents.

The development of novel seafood flavoring systems aligns with current trends in the food industry toward natural, clean-label ingredients that deliver authentic umami and marine complexity [19,20,21,22]. Concentrated seafood powders derived from high-value roe could provide a unique sensory foundation for soups, snacks, sauces, and premium food products; however, their functional performance depends heavily on how well freeze-drying preserves their chemical and organoleptic attributes. Evaluating freeze-dried roe and caviar as potential flavoring agents requires a comprehensive examination of nutritional retention, biochemical stability, microstructural changes, and sensory characteristics compared to their fresh counterparts.

Therefore, this study evaluates the feasibility of transforming blue crab roe, sea urchin roe, and Beluga caviar into premium, shelf-stable seafood flavorings through freeze-drying. By systematically characterizing their nutritional, lipid, and bioactive profiles alongside their in vitro antioxidant capacities, this research establishes the functional biochemical signatures of these distinct marine matrices. Furthermore, correlating GC-MS non-polar metabolite profiles with descriptive sensory and hedonic analyses provides crucial insights into how lyophilization shapes their organoleptic properties. Ultimately, these findings will guide the development of natural marine seasonings, expanding the culinary applications of luxury roes while promoting the sustainable valorization of invasive species.

## 2. Results

### 2.1. Physicochemical Characteristics and Nutritional Composition

The physicochemical parameters and nutritional profiles of the freeze-dried (FD) marine flavoring agents are summarized in Table 1. The freeze-drying process successfully yielded highly stable products, with water activity (aw) values ranging from 0.27 to 0.34, significantly below the threshold for microbial growth. FD beluga caviar exhibited the lowest aw (0.27), while FD blue crab roe and FD sea urchin showed similar values (approx. 0.33–0.34). The pH varied significantly between the matrices, with FD beluga caviar being the most alkaline (7.10) and FD sea urchin the most acidic (6.29). Regarding nutritional composition, FD blue crab roe presented the highest protein content (59.3%), significantly higher than both FD sea urchin and FD beluga caviar. Fat content was high in both FD blue crab roe (35.6%) and FD beluga caviar (33.2%), whereas FD sea urchin contained significantly less total fat (9.7%). Notably, FD beluga caviar contained the highest salt content (2.9%), reflecting its commercial “ready-to-eat” origin, followed by FD sea urchin (1.6%) and FD blue crab roe (0.7%). Carbohydrates and sugars were not detected in any of the final FD powders.

### 2.2. Total Polyphenol Content and Carotenoids

The concentration of bioactive compounds in the FD powders varied significantly among the three marine matrices (Table 2). FD blue crab roe was identified as the richest source of phenolic compounds (289.82 mg GAE/100 g), exceeding FD sea urchin by approximately 1.4 times and FD beluga caviar by 20 times. In terms of lipophilic pigments, FD sea urchin exhibited the highest total carotenoid concentration (121.29 mg/kg). FD blue crab roe contained approximately half the carotenoid content of the sea urchin (68.48 mg/kg). Conversely, FD beluga caviar exhibited minimal bioactive content across both phenolic and carotenoid measurements.

### 2.3. Antioxidant Capacity

**Figure 1:**
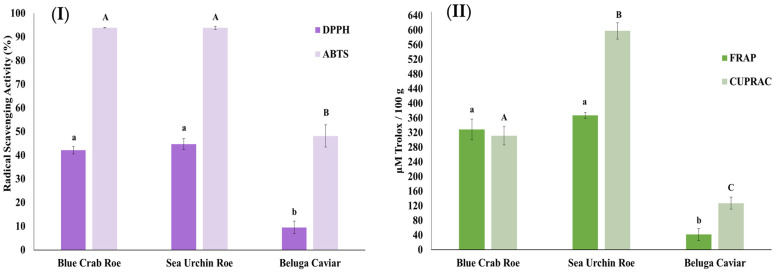
Antioxidant capacity of freeze-dried blue crab roe, sea urchin, and beluga caviar as determined by four complementary assays: (**I**) DPPH radical scavenging activity (%) and ABTS radical scavenging activity (%), (**II**) Ferric Reducing Antioxidant Power (FRAP) and Cupric Reducing Antioxidant Capacity (CUPRAC) expressed as μM Trolox equivalents/100 g. Data represent mean ± SD (*n* = 9). Different letters above the bars indicate statistically significant differences between the three freeze-dried products within each specific assay (*p* < 0.05). Lowercase letters (a, b) denote statistical comparisons for the DPPH and FRAP assays, while uppercase letters (A, B, C) denote comparisons for the ABTS and CUPRAC assays.

The antioxidant potential of the FD products was systematically evaluated using a multi-method approach involving four complementary assays (DPPH, ABTS, FRAP, and CUPRAC) to capture diverse chemical mechanisms of action, as varying reaction kinetics and solvent environments can significantly influence measured activity. Regarding radical scavenging activity, both FD blue crab roe and FD sea urchin exhibited exceptional performance in the ABTS assay, with inhibition levels exceeding 90% (Figure 1I). This was significantly higher than the scavenging activity recorded for FD beluga caviar, which reached only approximately 48%. A similar trend was observed in the DPPH assay, where FD blue crab roe and FD sea urchin demonstrated moderate yet superior inhibition, ranging between 42% and 45%, whereas FD beluga caviar showed significantly lower activity at approximately 9.5%.

The reducing power of the products, measured via FRAP and CUPRAC assays, further highlighted the biochemical advantage of the sea urchin and blue crab roe powders. FD sea urchin displayed the most robust reducing capacity among the three flavoring agents, particularly in the CUPRAC assay (597.88 μM Trolox/100 g) and FRAP assay (367.39 μM Trolox/100 g) (Figure 1II). FD blue crab roe followed closely in both assays, showing no statistically significant difference from the sea urchin in certain parameters. Conversely, FD beluga caviar consistently exhibited the lowest reducing potential across all tested assays, suggesting that its value as a flavoring agent is derived more from its unique lipid profile and sensory attributes than from its primary antioxidant capacity.

### 2.4. Fatty Acid Profile

The lipid profiles of the freeze-dried products revealed distinct nutritional signatures (Table 3). Regarding saturated fats, palmitic acid (C16:0) was identified as the predominant saturated fatty acid (SFA) across all samples (20–23%). However, the profiles diverged significantly in their unsaturated fractions. FD beluga caviar was uniquely characterized by a dominance of mono-unsaturated fatty acids (MUFAs), reaching 48.99% of the total fatty acids. This was driven by an exceptionally high concentration of oleic acid (C18:1n9c), which accounted for 43.1% of the profile. Additionally, the caviar sample contained 1.61% EPA and 6.62% DHA, achieving the highest desirable fatty acids (DFA) (77.57%) and the lowest Atherogenic Index (0.31).

In contrast, FD blue crab roe was characterized by a robust polyunsaturated fatty acid (PUFA) content (21.31%) and a highly favorable ω-6/ω-3 ratio of 0.46. The observed concentrations of EPA and DHA were 6.06% and 5.02%, respectively. Finally, FD sea urchin presented the lowest total PUFA content (9.66%), with significantly lower concentrations of EPA (0.11%) and DHA (0.12%). Instead, its profile showed a higher prevalence of saturated fats, resulting in higher Atherogenic Index (AI) (1.21) and Thrombogenic Index (TI) (1.24) values.

### 2.5. GC/MS Analysis

**Figure 2:**
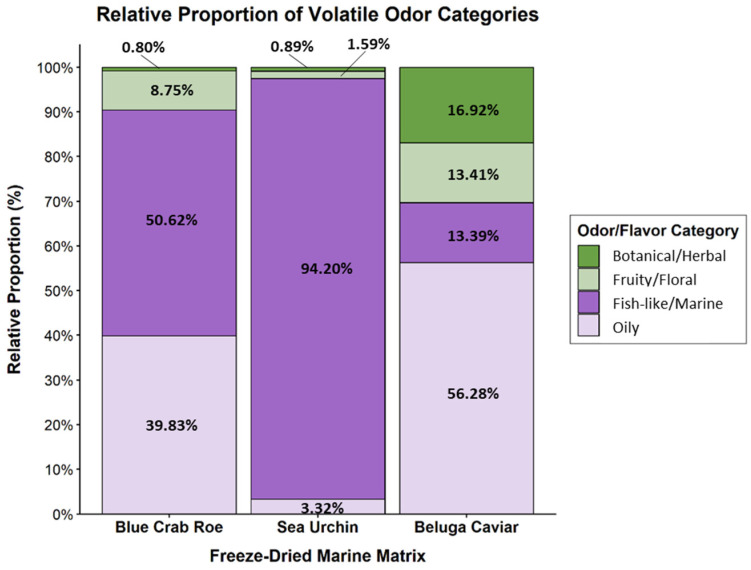
Relative proportion (%) of odor and flavor categories in freeze-dried Blue Crab Roe, Sea Urchin, and Beluga Caviar. The stacked bars illustrate the percentage contribution of four primary sensory classifications—Oily, Fish-like/Marine, Fruity/Floral, and Botanical/Herbal—to the total volatile profile of each marine matrix.

The non-polar and semi-volatile metabolites profiles of the three freeze-dried marine matrices were analyzed. Because even trace volatile compounds (<1%) can significantly contribute to macroscopic sensory perception, a comprehensive list of all identified compounds is provided in Supplementary Table S1. Also, representative Total Ion Chromatograms (TIC) demonstrating the separation quality and distinct volatile profiles of each freeze-dried marine matrix are provided in the Supplementary Materials (Figures S1–S3). Grouping these volatiles into primary sensory categories (Figure 2) revealed distinct compositional signatures. FD sea urchin roe was overwhelmingly dominated by “fish-like/marine” compounds (94.20%). FD blue Crab roe exhibited a more complex profile, primarily split between “fish-like/marine” (50.62%) and “oily” (39.83%) compounds. Conversely, FD beluga Caviar was characterized primarily by “oily” (56.28%) and “botanical/herbal” (16.92%) compounds, with the marine fraction comprising only 13.39% of its total area.

**Figure 3:**
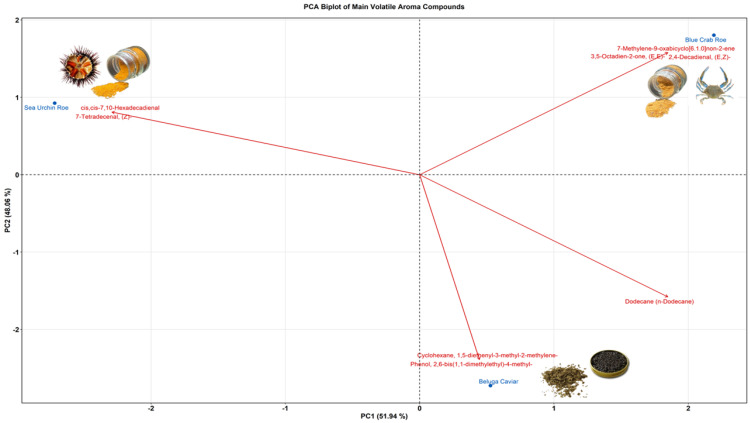
Principal Component Analysis biplot of the main volatile aroma compounds of Sea Urchin Roe, Blue Crab Roe, and Beluga Caviar. Blue points represent the sample scores, while red arrows represent the loadings of the main volatile aroma compounds, indicating the direction and magnitude of their contribution to the principal components.

To isolate the specific chemical markers driving this differentiation, a Principal Component Analysis (PCA) biplot of the most abundant compounds was generated (Figure 3). The first two principal components captured 100% of the variance (PC1: 51.94%; PC2: 48.06%), clearly separating the three matrices. The FD sea urchin profile was almost exclusively driven by two highly abundant marine aldehydes: 7-tetradecenal, (Z)- and cis, cis-7,10-hexadecadienal. FD blue crab roe was characterized by a distinct mix of aldehydes and ketones, including 7-methylene-9-oxabicyclo[6.1.0]non-2-ene, (E,E)-3,5-Octadien-2-one, and (E,Z)-2,4-decadienal reflecting its fatty notes. Finally, FD beluga caviar separated based on its heavier hydrocarbons and phenolic compounds, specifically dodecane (n-dodecane), 2,6-bis(1,1-dimethylethyl)-4-methyl-phenol and 1,5-diethenyl-3-methyl-2-methylene-cyclohexane, which directly align with its dominant oily and herbal sensory categorizations.

### 2.6. Sensory Evaluation

To determine the organoleptic feasibility and consumer reception of the FD marine flavoring agents, a comprehensive sensory evaluation was conducted. This assessment combined descriptive profiling of texture, flavor, and aroma with a hedonic analysis to measure overall consumer acceptability.

**Figure 4:**
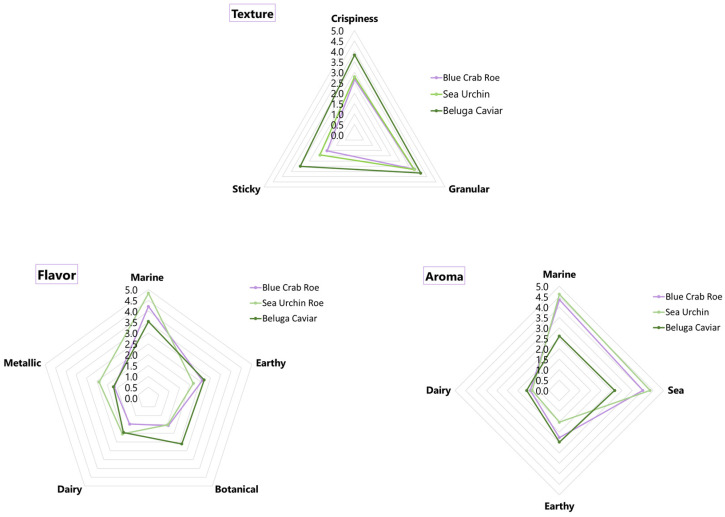
Graphical representation of the qualitative descriptive sensory profiles (Texture, Flavor, and Aroma) for the freeze-dried marine flavoring agents.

The descriptive radar charts revealed highly distinct sensory signatures for each marine matrix (Figure 4). Texturally, FD beluga caviar dominated, scoring the highest for granularity, stickiness, and crispiness, whereas FD blue crab roe and FD sea urchin presented much milder, less granular, and less sticky textures. In terms of flavor and aroma, FD sea urchin and FD blue crab roe were strongly characterized by intense “marine” and “sea” notes, with the sea urchin also exhibiting distinct metallic flavors. Conversely, FD beluga caviar displayed a significantly milder marine profile, distinguished instead by prominent earthy and dairy aromatic and flavor notes.

**Figure 5:**
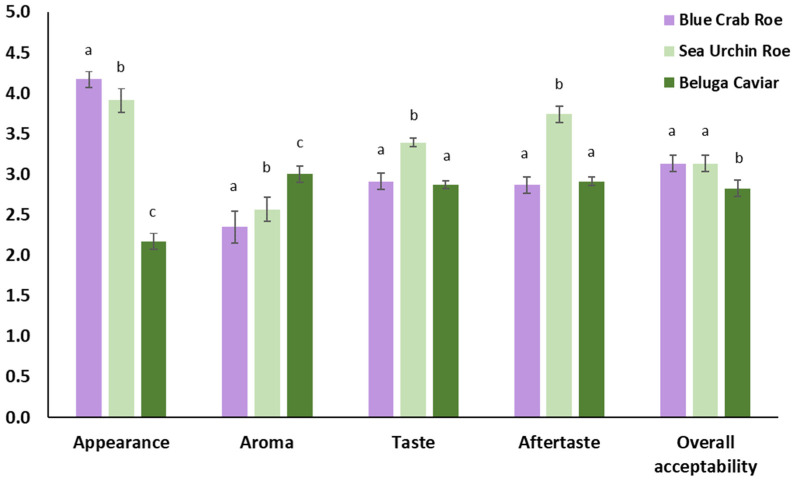
Consumer acceptance of different attributes for the freeze-dried blue crab roe, sea urchin, and beluga caviar. Different lowercase letters above the bars within the same sensory attribute indicate statistically significant differences between the three marine matrices (*p* < 0.05) by Tukey’s test.

These descriptive characteristics directly translated into the consumer acceptability scores, which were evaluated on a 5-point hedonic scale (Figure 5). For appearance, FD blue crab roe and FD sea urchin were highly rated (scoring approximately 4.2 and 3.9, respectively), significantly outperforming the FD beluga caviar (2.2). While Beluga caviar scored the highest in the aroma category (3.0), FD sea urchin roe emerged as the most preferred in terms of taste (3.4) and aftertaste (3.8). Ultimately, both FD blue crab roe and FD sea urchin achieved the highest and statistically equivalent overall acceptability scores (3.1), indicating a positive consumer reception, while FD beluga caviar received a slightly lower overall rating (2.8) as a powdered flavoring agent.

## 3. Discussion

### 3.1. Physicochemical Stability and Nutritional Composition

The physicochemical parameters confirmed the efficacy of the freeze-drying process. The low aw values (0.27–0.34) confirm that the freeze-drying process was highly effective. These levels are well below the 0.60 threshold required to stop microbial growth, ensuring the powders are shelf-stable without preservatives [23]. While there is a lack of existing data specifically for freeze-dried marine roe powders, our results show that this method could successfully preserve the samples’ biochemical integrity, as evidenced by the stable pH levels. Nutritionally, freeze-drying concentrated the raw materials into high-density ingredients. The high protein content in blue crab roe (59.3%) is especially noteworthy, as it proves that invasive species can be transformed into premium, nutrient-dense resources [24]. The high fat levels in the crab roe and caviar act as excellent carriers for flavor. Although the Beluga caviar has a higher salt content (2.9%), this is expected for a “ready-to-eat” product and actually benefits its use as a gourmet seasoning. These findings highlight the potential of these powders as “clean-label” and high-protein flavoring agents.

### 3.2. Total Polyphenols and Carotenoids

The high phenolic content observed in the FD blue crab roe is particularly noteworthy, given that quantitative data on polyphenols in marine animal tissues are generally limited compared to marine algae [25]. This elevated phenolic concentration likely results from bioaccumulation through the crab’s diet, specifically from the consumption of phytoplankton and algae, thereby enhancing the functional potential of the roe as an antioxidant-rich flavoring agent. Regarding lipophilic pigments, the high carotenoid concentration found in FD sea urchin aligns closely with previously reported ranges for *Paracentrotus lividus* (103–114 mg/kg), where specific carotenoids such as echinenone and β-carotene are known to be dominant [26,27]. Although the FD blue crab roe contained fewer total carotenoids than the sea urchin, it remains a substantial source of astaxanthin, the primary pigment responsible for its characteristic reddish hue [20,28]. Finally, the minimal bioactive content observed in the FD beluga caviar confirms that its functional and nutritional value is derived primarily from its high protein and lipid profile rather than its antioxidant constituents.

### 3.3. Antioxidant Capacity

The antioxidant capacity of the freeze-dried marine matrices varied significantly across the four complementary assays evaluated (DPPH, ABTS, FRAP, and CUPRAC), reflecting their diverse functional potentials. These variations in antioxidant performance are strongly correlated with the specific bioactive profiles of the matrices. FD blue crab roe emerged as the richest source of phenolic compounds (289.82 mg GAE/100 g), a finding that directly explains its superior performance in radical scavenging assays. Specifically, the strong relationship observed between high TPC and elevated inhibition in DPPH and ABTS assays is consistent with the findings of Salar and Purewal (2016) [29] and Arivalagan et al. (2018) [30]. These studies reported high Pearson’s correlation coefficients between TPC and antioxidant activity across all four assays, confirming that phenolic compounds are major contributors to the scavenging of free radicals in complex food matrices.

In the case of FD sea urchin, the antioxidant mechanism appears to be driven by its unique pigment profile in addition to phenolics. The sample exhibited the highest total carotenoid concentration (121.29 mg/kg) and achieved the highest reducing power values. The CUPRAC and FRAP assays are particularly effective for assessing the reducing capability of such bioactive extracts, as demonstrated by Alafnan et al. (2021) [31] and Tousif et al. (2023) [32]. These researchers noted that extracts rich in secondary metabolites often display superior activity in reducing power assays compared to radical scavenging ones, validating the specific potency of the sea urchin’s lipophilic antioxidants.

The overall high antioxidant retention across the blue crab and sea urchin samples can be attributed to the freeze-drying process itself. Shahimoridi et al. (2025) [33] and Ma et al. (2024) [34] have documented that freeze-drying preserves significantly higher levels of phenolics and antioxidant capacity, especially in FRAP and DPPH assays, compared to thermal drying methods, which often degrade heat-sensitive bioactive components. Belayneh Asfaw et al. (2024) [35] further support this, noting that lyophilization enhances the recovery of TPC and subsequent antioxidant activity in biological tissues. In contrast, the minimal activity observed in FD Beluga caviar confirms that its functional value lies in its nutritional composition (lipids/proteins) rather than as an oxidation inhibitor.

### 3.4. Fatty Acid Profile

The distinct lipid profiles define the potential applications of these matrices as functional food ingredients. The dominance of oleic acid in FD beluga caviar aligns with the findings of Ghelichi et al. (2022) [36] and Caprino et al. (2008) [37], who identified it as the major MUFA in sturgeon roe, contributing to its characteristic “melting” mouthfeel and premium sensory quality. Although Bekhit et al. (2022) [38] reported slightly higher EPA values (2.89%) for beluga caviar compared to our FD sample, the DHA content remains comparable to the 8.45% reported in the literature, confirming that freeze-drying successfully preserves these essential lipids. This positions the beluga caviar powder as a source of heart-healthy lipids comparable to high-quality marine oils.

For FD blue crab roe, the robust PUFA profile and high EPA/DHA concentrations are consistent with the ranges reported by Çelik et al. (2004) [39] for blue crab tissues. Wu et al. (2020) [40] and Naczk et al. (2004) [41] similarly highlighted that crab gonads are critical reservoirs for these PUFAs, which are essential for cardiovascular health. The successful preservation of these oxidation-sensitive fatty acids in the freeze-dried powder supports the argument for valorizing invasive species, effectively transforming an environmental burden into a stable, nutrient-dense ingredient.

Conversely, the FD sea urchin lipid profile deviated from the typical high-PUFA values often cited in the literature, such as the rich sources described by Ciriminna et al. (2024) [42] and Wang et al. (2022) [43]. This variation is likely attributable to the specific diet and harvest season of the urchins used, as Verachia et al. (2022) [10] and Baião et al. (2019) [44] emphasize that the lipid composition of *Paracentrotus lividus* is highly plastic and significantly influenced by its macroalgal feed. Despite the lower ω-3 content compared to the other matrices, the presence of unique fatty acids alongside the high carotenoid content discussed previously suggests that the true functional value of the sea urchin powder lies in its complex bioactive pigment-lipid matrix rather than its fatty acid profile alone.

### 3.5. GC-MS Analysis

The GC-MS analysis of the non-polar and semi-volatile metabolites revealed highly distinct chemical signatures that define the unique aroma profile and oxidative state of each freeze-dried matrix.

For the FD blue crab roe, the dominant ketones and aldehydes corroborate the findings of Du et al. (2025) [45], who noted that lipid peroxidation shifts the fresh ester profile toward the heavy, oily flavor constituents observed in FD blue crab roe.

Conversely, the FD beluga caviar deviated from the typical “sea-fresh” and “buttery” aromas widely reported for commercial caviar [46,47]. Its unusually low marine fraction and prominent heavy hydrocarbons and phenolics align more closely with oxidized profiles, where processing replaces fresh notes with earthy and oily off-flavors [48,49]. This highlights the severe impact of the freeze-drying process on the oxidative stability of this lipid-dense matrix, presenting a stark contrast to the better-preserved sensory profiles often observed in differently processed caviar studies [7].

Finally, the FD sea urchin exhibited an overwhelmingly pure marine aldehyde signature, presenting a much narrower profile than the multi-faceted odors typically documented across different urchin populations [50]. This highly concentrated seafood aroma underscores its potential as a potent marine flavoring agent prior to further formulation [51].

### 3.6. Correlation of Chemical Profiles with Sensory Analysis

The sensory reception of the freeze-dried marine matrices can be directly explained by the interaction between their fatty acid composition and their resulting flavor or odor identified metabolites (GC-MS). The degradation, preservation, and oxidation of specific lipid fractions during processing ultimately dictated the macroscopic flavor and aroma profiles perceived by the panelists.

The FD sea urchin achieved the highest consumer scores for taste and aftertaste, alongside a top overall acceptability rating (3.1). The FAMEs analysis revealed that this matrix contained the lowest total PUFA content (9.66%). Because it lacked the highly unstable lipid fractions that typically cause rapid rancidity, it did not develop oxidized off-flavors during freeze-drying. Instead, it yielded a highly pure, concentrated volatile profile consisting of 94.20% “fish-like/marine” aldehydes (specifically (Z)-7-tetradecenal and cis,cis-7,10-hexadecadienal). This pure chemical signature perfectly correlates with the intense, clean “marine” and “sea” notes identified in the descriptive sensory profiling, explaining its high consumer preference.

Similarly, the FD blue crab roe achieved top scores in overall acceptability (3.1) and was characterized by a complex, rich sensory profile. The FAMEs data showed a robust concentration of PUFAs (21.31%), which are highly susceptible to lipid peroxidation. The GC-MS data confirm this oxidation occurred, but in a highly favorable manner: the degradation of these fatty acids generated pleasant, flavor-enhancing ketones and aldehydes (3,5-octadien-2-one and 2,4-decadienal). This chemical shift split its volatile profile evenly between marine (50.62%) and oily/fatty (39.83%) categories. The sensory panel directly detected this biochemical complexity, rating the crab roe highly for strong “marine” notes layered with a rich, savory mouthfeel, proving that controlled lipid oxidation in crustacean gonads can generate highly desirable flavoring agents.

In contrast, the FD beluga caviar received the lowest scores for taste (2.8) and overall acceptability (2.8). The caviar was exceptionally rich in total fat (33.2%) and dominated by MUFAs (48.99%, primarily oleic acid). This heavy lipid matrix underwent significant degradation during processing, resulting in a GC-MS profile completely dominated by heavy hydrocarbons (n-dodecane) and phenolics, while its fresh marine fraction was reduced to just 13.39%. The sensory panel’s evaluation perfectly mirrored this chemical shift as they scored the caviar lowest in fresh “marine/sea” attributes and highest in “earthy” and “dairy” descriptors, indicating a loss of fresh seafood character. Furthermore, the combination of high fat and high salt (2.9%) physically compromised the powder, resulting in the high “granular” and “sticky” texture scores that detracted from its sensory appeal.

While this study provides a comprehensive biochemical, nutritional, and sensory characterization of the freeze-dried marine matrices, certain limitations must be acknowledged. The present work focused primarily on the chemical and oxidative stability of the biomolecules; however, it did not evaluate the physical and microstructural properties of the resulting powders. Parameters such as particle size distribution, porosity, and surface morphology were not determined, although they play a critical role in the technological functionality of food powders, particularly regarding rehydration kinetics, solubility, and flowability. Future studies should integrate these microstructural analyses alongside targeted physical and textural optimization strategies to fully evaluate the behavior and stability of these freeze-dried marine powders in commercial food formulations.

## 4. Materials and Methods

### 4.1. Samples

Blue crab roe (*Callinectes sapidus*) and sea urchin roe (*Paracentrotus lividus*) were kindly provided by Forkys Food Company (Thessaloniki, Greece), while Beluga caviar (*Huso huso*) was purchased from the market. The blue crab roe was manually removed following the industrial steaming of the whole crabs. The sea urchin gonads and beluga caviar were utilized in their commercial ‘ready-to-eat’ form. All samples were immediately frozen at −20 °C for 24 h as a necessary pre-treatment step prior to freeze-drying (as detailed in Section 4.2).

### 4.2. Preparation of Samples and Freeze-Drying Parameters

To produce the freeze-dried (FD) products, all the samples were initially stored at −20 °C for 24 h to ensure the complete conversion of water into ice. Subsequently, the frozen samples were transferred to a laboratory freeze-dryer (Telstar Lyoquest, Barcelona, Spain). The process was conducted at a condenser temperature of −55 °C and a vacuum pressure of 0.1 mbar until full sublimation of the ice was achieved, resulting in dehydrated matrices. Following the completion of the freeze-drying cycle, the samples were pulverized using a laboratory blender to obtain a fine powder with a uniform particle size.

### 4.3. Physicochemical Analysis

The pH of all samples was determined in a 5% (*w*/*v*) aqueous solution using a Consort C5010 pH meter (Concort bvba, Turnhout, Belgium), ensuring precise and reliable measurements. Additionally, water activity (aw) was measured using a specialized water activity meter (Aqualab Pawkit, Meter Group, München, Germany) according to the manufacturer’s instructions. The nutritional composition of the freeze-dried samples was determined according to standard AOAC official methods [52]. Specifically, total protein content was evaluated using the Kjeldahl method (using a nitrogen-to-protein conversion factor of 6.25). Total fat was extracted and quantified using the Soxhlet method, while NaCl content was determined via volumetric titration. Finally, total carbohydrates were calculated by difference: 100 − (% protein + % fat + % ash + % moisture).

### 4.4. Extraction of Bioactive Compounds

The extraction of bioactive compounds was performed according to the method described by Miraglia et al. (2020) [53] with minor modifications. Specifically, 1 g of each freeze-dried sample was extracted with 5 mL of an 80% (*v*/*v*) aqueous methanol solution. The mixtures were vortexed and then placed in an ultrasonic bath (Branson 2510, Emerson, Brookfield, CT, USA) for 10 min. Following ultrasonication, the samples were subjected to mechanical stirring at 400 rpm for 1 h at 25 °C. After stirring, the samples were centrifuged (Eppendorf Centrifuge 5702R, Hamburg, Germany) at 4000 rpm for 10 min. The supernatant was carefully collected, and the extraction procedure was repeated twice. The combined supernatants were evaporated to dryness under vacuum using a centrifugal concentrator (Eppendorf SpeedVac, Concentrator 5501, Hamburg, Germany). The resulting dry residue was reconstituted in 5 mL of 100% methanol. To ensure complete dissolution, the samples were vortexed and placed in an ultrasonic bath until the residue was fully dissolved. Finally, the extracts were centrifuged at 4000 rpm for 10 min and filtered through 0.45 μm PVDF filters. The final extracts were used for the determination of total polyphenol content (TPC) and antioxidant capacity.

### 4.5. Total Polyphenol Content (TPC)

Total phenolic content was determined using the Folin–Ciocalteu assay according to the method of Ha et al. (2022) [54], with minor modifications. The standard curve was created using gallic acid (20–500 ppm) as the reference solution. For the assay, 50 μL of sample extract was mixed with 600 μL of deionized water, 50 μL of Folin–Ciocalteu reagent (Sigma-Aldrich, St. Louis, MO, USA), and 300 μL of 20% (*w*/*v*) sodium carbonate (Na_2_CO_3_) solution. The samples were incubated in a dark place at room temperature for 1 h, followed by centrifugation at 3000× *g* for 10 min. Absorbance was measured at 725 nm, and the total polyphenol content is expressed as mg of gallic acid equivalent (GAE) per 100 g of sample.

### 4.6. Determination of Antioxidant Capacity

#### 4.6.1. DPPH Radical Scavenging Activity

The DPPH assay was performed according to Duan et al. (2007) [55] and Ha et al. (2022) [54] with minor modifications. Briefly, 25 μL of the extract was mixed with 975 μL of a 0.1 mM DPPH methanolic solution (Sigma, USA). The mixture was vortexed and incubated in the dark at room temperature for 30 min. The absorbance was measured at 515 nm using a Shimadzu UV-2600 spectrophotometer (Shimadzu Corporation, Kyoto, Japan). The radical scavenging activity was calculated as the percentage of DPPH neutralization using the following equation:

Radical Scavenging Activity (%) = [(A_0_ − A)/A_0_] × 100 (1)

where A_0_ is the absorbance of the control and A is the absorbance of the sample.

#### 4.6.2. ABTS Radical Scavenging Activity

The ABTS assay was performed according to Loganayaki et al. (2013) [56]. A stock solution was prepared by mixing 7 mM ABTS and 2.5 mM K_2_S_2_O_8_ (1:1 *v*/*v*) and incubating for 16–18 h in the dark. Briefly, 50 μL of sample extract was added to 950 μL of the ABTS solution, vortexed, and incubated in the dark at room temperature for 30 min. Absorbance was measured at 734 nm and the antioxidant capacity was calculated using Equation (1).

#### 4.6.3. Ferric Reducing Antioxidant Power (FRAP) Assay

The FRAP assay was conducted following the method of Powell et al. (2014) [57]. The FRAP reagent was prepared by mixing 0.3 M acetate buffer (pH 3.6), 0.01 M TPTZ in 0.04 M HCl, and 0.02 M FeCl_3_·6H_2_O in a 10:1:1 ratio. Briefly, 50 μL of sample extract was mixed with 1.45 mL of FRAP reagent, incubated at 37 °C for 10 min, and then cooled to room temperature. Absorbance was measured at 593 nm using a Shimadzu UV-2600 spectrophotometer and the results were calculated against a Trolox standard curve (10–1000 μM) and expressed as μM Trolox equivalents per 100 g of sample.

#### 4.6.4. Cupric Reducing Antioxidant Capacity (CUPRAC) Assay

The CUPRAC assay was performed according to Apak et al. (2004) [58]. In a 2 mL tube, 100 μL of sample extract was mixed with 200 μL each of 10 mM CuCl_2_, 7.5 mM neocuproine in ethanol, and 1 M NH_4_Ac. The mixture was incubated at room temperature for 30 min, and absorbance was measured at 450 nm. Antioxidant capacity was determined via a Trolox standard curve (10–1000 μM) and expressed as μM Trolox equivalents per 100 g of sample.

### 4.7. Extraction and Quantification of Total Carotenoids

Total carotenoid extraction was performed according to the method of Hooshmand et al. (2017) [59]. Briefly, 1 g of freeze-dried samples were subjected to repeated extraction with 5 mL of acetone until the extract became colorless. The acetonic extracts were combined and subsequently filtered through a 0.22 μm Millipore filter and dehydrated using sodium sulfate (Na_2_SO_4_). As astaxanthin was identified as the predominant carotenoid and preliminary spectrophotometric analysis (Shimadzu UV-2600) showed maximum absorbance at 478 nm, all measurements were conducted at this wavelength. Total carotenoid concentration was calculated using the following equation:

Total Carotenoids (mg/kg) = (A_478_ × V)/(0.22 × W) (2)

where A_478_ is the absorbance at 478 nm, V is the total volume of the extract (mL), 0.22 is the absorbance of a 1 mg/L astaxanthin standard solution in acetone (1 cm path length), and W is the sample weight (g).

### 4.8. Determination of Fatty Acid Composition (FAMEs)

Fatty acids were extracted and determined according to the direct FAME synthesis method described by O’Fallon et al. (2007) [60]. Briefly, 1 g of sample was subjected to alkaline hydrolysis with 10 N methanolic KOH with 0.01% BHT at 55 °C for 90 min, followed by acid-catalyzed methylation with 24 N H_2_SO_4_ at 55 °C for an additional 90 min. The resulting fatty acid methyl esters (FAMEs) were extracted into hexane and collected after centrifugation (4 °C, 5 min, 2500 rpm). Fatty acid composition was determined using a GCMS-QP2010 Ultra Gas chromatograph mass spectrometer (Shimadzu Europe, GmbH, Duisburg, Germany) equipped with an SP-2340 capillary column (100 m × 0.25 mm, 0.20 μm) (Supelco, Bellefonte, PA, USA). The injector and detector temperatures were set at 250 °C. The oven temperature program started at 100 °C for 5 min, increased to 240 °C at a rate of 4 °C/min, and was held for 30 min. Helium was used as the carrier gas at a flow rate of 20 cm/min. Qualitative identification of individual fatty acids was achieved by comparing the retention times of the detected peaks in the samples with those of the authentic standards present in the commercial mixture Supelco 37 Component FAME Mix (Sigma-Aldrich) analyzed under identical chromatographic conditions. Quantitative evaluation was performed using the peak area normalization method; specifically, the relative proportion of each fatty acid was calculated by expressing its individual integrated peak area as a percentage of the total peak area of all identified FAMEs in the chromatogram.

### 4.9. GC/MS Analysis

To profile the non-polar and semi-volatile metabolites responsible for flavor and odor characteristics, the FD samples were subjected to a liquid–liquid solvent extraction prior to GC-MS analysis [61]. Approximately 1 g of the homogenized FD powder was extracted with 4 mL of a hexane:diethyl ether (90:10, *v*/*v*) mixture and vigorously vortexed for 1 min. The mixture was then centrifuged for 2 min at 20,238× *g* to facilitate phase separation. The non-polar supernatant was carefully collected, dehydrated over anhydrous sodium sulfate to remove any residual moisture, and subsequently filtered through a PTFE syringe filter (0.45 μm pore size, 25 mm diameter). To concentrate the semi-volatile compounds, the filtered extract was reduced to a final volume of 0.2 mL under a gentle stream of nitrogen gas.

The concentrated extracts were analyzed using a GC-2010 Plus Shimadzu gas chromatograph (Shimadzu Europe, GmbH) coupled with a GCMS-QP2010 Ultra mass spectrometer (Shimadzu Europe, GmbH), equipped with a MEGA-5MS capillary column (30 m length × 0.25 mm internal diameter, 0.25 μm film thickness). Injections were performed in splitless mode. The temperatures of the injector and the detector were set to 250 °C and 300 °C, respectively. The oven temperature program was initiated at 60 °C, increased at a rate of 3 °C/min to 240 °C, and held isothermally for 5 min to ensure equilibration. Subsequently, the temperature was raised at a rate of 10 °C/min to 290 °C and maintained for an additional 10 min to facilitate the elution of compounds with higher boiling points. Helium was utilized as the carrier gas at a constant flow rate of 1.3 mL/min.

Mass spectra were acquired in scan mode. The qualitative identification of the metabolites was performed by comparing the generated mass spectra against established reference libraries, including the FFNSC GC/MS Ver. 1.3, the Shimadzu Metabolite Component Database, Wiley 7, NIST 11, and NIST 11s. To ensure a high degree of analytical confidence, only compounds demonstrating a mass spectral similarity match score of strictly >80% across these libraries were accepted for inclusion in the final profiling. Because the primary objective of this analysis was the comparative profiling of sensory groups across different matrices, the volatile compound data are expressed as relative percentage areas rather than absolute or semi-quantitative concentrations.

### 4.10. Sensory Evaluation

To assess their potential application as high-value flavoring agents in culinary and industrial food formulations, the sensory characteristics of the freeze-dried blue crab roe, sea urchin, and beluga caviar were systematically evaluated. The analysis aimed to determine the intensity of specific attributes that define the quality and commercial viability of these developed powders. The sensory panel consisted of 25 assessors by the Institute of Applied Biosciences of CERTH. Ethical protocols were strictly followed to ensure participant privacy and rights; informed written consent was obtained from all volunteers, who were free to withdraw from the study at any time without penalty. For the evaluation Quantitative Descriptive Analysis (QDA) was used. Briefly, an unstructured 5-point scale was employed (where 1 = not perceptible/extremely poor and 5 = extremely intense/excellent). Panelists evaluated the products based on 25 predefined descriptive terms regarding appearance, odor, taste, aroma, texture/mouthfeel, and aftertaste, as established by Phillips et al. (2010) [6]. Finally, the overall impression and preference ranking were recorded for each product. Samples (2 g each) were presented in a randomized order using 3-digit blind codes to eliminate carry-over effects [62]. Between samples, panelists neutralized their palate using salt-free, sugar-free bread and mineral water.

### 4.11. Statistical Analysis

Three independent batches of each marine matrix were processed as biological replicates, and each was analyzed in triplicate (technical replicates), yielding nine total observations (*n* = 9) per assay. Data are expressed as mean values ± standard deviation, calculated by pooling all replicate data to reflect total sample variance. Initial data processing and organization were carried out using Microsoft Office Excel. Statistical significance was determined using a one-way analysis of variance (ANOVA) followed by an appropriate post hoc test (GraphPad Prism 11.0.0, San Diego, CA, USA). Differences were considered statistically significant at a *p*-value threshold of ≤0.05. Additionally, advanced data visualization and multivariate statistical analyses, including Principal Component Analysis (PCA), were executed using R software within the RStudio 4.3.1 environment.

## 5. Conclusions

In conclusion, the sensory quality and oxidative stability of freeze-dried marine powders are fundamentally dictated by the synergy between their initial lipid composition and endogenous antioxidant capacity. Matrix-specific responses varied significantly: FD sea urchin (high carotenoids/low PUFA) maintained exceptional stability and pure marine flavor, whereas FD blue crab roe leveraged high phenolics to buffer its robust PUFAs into desirable savory notes. In contrast, the high-fat, low-antioxidant FD beluga caviar suffered severe oxidative degradation and off-flavors. While these baseline biochemical profiles prove the strong commercial potential of valorizing species like the invasive blue crab into clean-label flavoring agents, optimizing their industrial application requires further investigation. To fully realize this potential, future research must prioritize microstructural and textural optimization, alongside prolonged storage trials, to establish the technological functionality, long-term shelf-life stability, and ultimate commercial viability of these novel marine powders.

## Figures and Tables

**Table 1:** pH, water activity (aw) and nutritional composition of freeze-dried (FD) blue crab roe, sea urchin and beluga caviar. Values are expressed as mean ± SD (*n* = 9). Different lowercase letters within the same row indicate statistically significant differences between the different freeze-dried powders (*p* < 0.05).

Parameters	FD Blue Crab Roe	FD Sea Urchin	FD Beluga Caviar
pH	6.48 ± 0.01 ^a^	6.29 ± 0.01 ^b^	7.10 ± 0.01 ^c^
aw	0.33 ± 0.01 ^a^	0.34 ± 0.00 ^a^	0.27 ± 0.01 ^b^
Protein (%)	59.3 ± 1.25 ^a^	46.6 ± 1.96 ^b^	48.5 ± 1.65 ^b^
Fat (%)	35.6 ± 1.00 ^a^	9.7 ± 0.35 ^b^	33.2 ± 1.15 ^a^
Saturated fat (%)	7.2 ± 0.25 ^a^	1.9 ± 0.1 ^b^	8.9 ± 0.1 ^c^
Carbohydrates (%)	0 ± 0.00	0 ± 0.00	0 ± 0.00
Sugars (%)	0 ± 0.00	0 ± 0.00	0 ± 0.00
Salt (%)	0.7 ± 0.05 ^a^	1.6 ± 0.10 ^b^	2.9 ± 0.20 ^c^

**Table 2:** Total polyphenol and carotenoid concentrations of freeze-dried (FD) blue crab roe, sea urchin and beluga caviar. Values are expressed as mean ± SD (*n* = 9). Values are expressed as mean ± SD (*n* = 9). Different lowercase letters within the same row indicate statistically significant differences between the different freeze-dried powders (*p* < 0.05).

FD Products	Total Polyphenol (mg GAE/100 g)	Total Carotenoids (mg/Kg)
FD Blue crab roe	289.82 ± 8.69 ^a^	68.48 ± 0.13 ^a^
FD Sea urchin roe	201.70 ± 4.48 ^b^	121.29 ± 0.13 ^b^
FD Beluga caviar	14.31 ± 1.28 ^c^	5.38 ± 0.13 ^c^

**Table 3:** Fatty acid composition (%) of freeze-dried (FD) blue crab roe, sea urchin and beluga caviar.

Fatty Acid (%)	FD Blue Crab Roe	FD Sea Urchin	FD Beluga Caviar
C10:0	0.08 ± 0.04 ^a^	0.38 ± 0.04 ^b^	0.17 ± 0.00 ^c^
C12:0	0.15 ± 0.01 ^a^	0.29 ± 0.12 ^a^	ND
C14:0	4.02 ± 0.24 ^a^	5.87 ± 0.22 ^b^	0.78 ± 0.03 ^c^
C14:1	0.32 ± 0.04 ^a^	0.59 ± 0.12 ^b^	ND
C15:0	0.87 ± 0.02 ^b^	1.05 ± 0.05 ^b^	0.20 ± 0.01 ^c^
C15:1	0.32 ± 0.07	ND	ND
C16:0	23.5 ± 1.45 ^a^	23.72 ± 0.32 ^a^	20.27 ± 0.19 ^b^
C16:1	20.95 ± 0.81 ^a^	14.42 ± 0.66 ^b^	4.00 ± 0.13 ^c^
C17:0	2.21 ± 0.03 ^a^	2.61 ± 0.11 ^b^	0.32 ± 0.04 ^c^
C17:1	1.30 ± 0.12 ^a^	1.00 ± 0.14 ^b^	0.32 ± 0.01 ^c^
C18:0	5.86 ± 0.07 ^a^	5.04 ± 0.09 ^b^	2.44 ± 0.11 ^c^
C18:1n9t	1.39 ± 0.08 ^a^	1.40 ± 0.16 ^a^	0.28 ± 0.04 ^b^
C18:1n9c	12.25 ± 0.55 ^a^	7.93 ± 0.1 ^b^	43.1 ± 0.28 ^c^
C18:2n6t	0.36 ± 0.07 ^a^	0.48 ± 0.04 ^a^	ND
C18:2n6c	2.08 ± 0.24 ^a^	2.61 ± 0.02 ^b^	10.74 ± 0.34 ^c^
C20:0	0.38 ± 0.08 ^ab^	0.54 ± 0.09 ^a^	0.38 ± 0.02 ^b^
C18:3n6	0.29 ± 0.1 ^a^	0.90 ± 0.14 ^b^	1.75 ± 0.06 ^c^
C20:1n9	4.05 ± 0.01 ^a^	3.65 ± 0.01 ^b^	1.12 ± 0.05 ^c^
C18:3n3	1.71 ± 0.06 ^ab^	2.18 ± 0.36 ^a^	1.59 ± 0.07 ^b^
C21:0	ND	0.86 ± 0.81	ND
C20:2	0.66 ± 0.09 ^a^	1.62 ± 0.08 ^b^	0.59 ± 0.03 ^a^
C22:0	0.35 ± 0.06 ^a^	0.19 ± 0.07 ^b^	0.31 ± 0.05 ^a^
C20:3n6	0.35 ± 0.07 ^a^	0.35 ± 0.00 ^a^	0.50 ± 0.01 ^b^
C22:1n9	0.29 ± 0.05 ^a^	0.57 ± 0.00 ^b^	0.16 ± 0.03 ^c^
C20:3n3	1.79 ± 0.15 ^a^	0.74 ± 0.15 ^b^	0.18 ± 0.00 ^c^
C20:4n6	2.50 ± 0.07 ^a^	0.44 ± 0.44 ^b^	2.24 ± 0.08 ^c^
C22:2	0.49 ± 0.19 ^a^	0.10 ± 0.10 ^b^	0.32 ± 0.01 ^a^
C20:5n3	6.06 ± 0.04 ^a^	0.11 ± 0.11 ^b^	1.61 ± 0.01 ^c^
C24:1n9	0.40 ± 0.18 ^a^	0.03 ± 0.03 ^b^	ND
C22:6n3	5.02 ± 0.11 ^a^	0.12 ± 0.02 ^b^	6.62 ± 0.09 ^c^
ω-3	14.57 ± 0.20 ^a^	3.16 ± 0.42 ^b^	10.01 ± 0.11 ^c^
ω-6	6.73 ± 0.47 ^a^	6.50 ± 0.48 ^a^	16.13 ± 0.36 ^b^
ω-9	35.27 ± 1.00 ^a^	24.36 ± 0.69 ^b^	47.54 ± 0.31 ^c^
ω-6/ω-3	0.46 ± 0.03 ^a^	2.06 ± 0.03 ^b^	1.61 ± 0.03 ^c^
MCFAs	5.13 ± 0.24 ^a^	7.60 ± 0.26 ^b^	1.15 ± 0.32 ^c^
MUFAs	41.28 ± 1.01 ^a^	29.60 ± 0.71 ^b^	48.99 ± 0.32 ^a^
PUFAs	21.31 ± 0.51 ^a^	9.66 ± 0.64 ^b^	26.13 ± 0.37 ^c^
DFAs	68.44 ± 1.13 ^a^	44.30 ± 0.96 ^b^	77.57 ± 0.50 ^c^
AI	0.63 ± 0.02 ^a^	1.21 ± 0.02 ^b^	0.31 ± 0.01 ^c^
TI	0.48 ± 0.01 ^a^	1.24 ± 0.04 ^a^	0.37 ± 0.01 ^a^

Values are expressed as mean ± SD (*n* = 9). Values in the same row with different letters differed significantly (*p* < 0.05). Notations as in Table 1. ND: Not Detected; Medium chain fatty acids (MCFAs); Monounsaturated fatty acids (MUFAs); Polyunsaturated fatty acids (PUFAs); Desirable fatty acids (DFAs); Atherosclerosis Index (AI), Thrombogenicity Index (TI).

## Data Availability

All data generated or analyzed during this study are included in this article.

## Supplementary Materials

The following supporting information can be downloaded at https://www.mdpi.com/article/10.3390/md24040135/s1, Figure S1. Representative Total Ion Chromatograms (TIC) of the volatile compounds identified in the freeze-dried sea urchin. Figure S2. Representative Total Ion Chromatograms (TIC) of the volatile compounds identified in the freeze-dried beluga caviar. Figure S3. Representative Total Ion Chromatograms (TIC) of the volatile compounds identified in the freeze-dried blue crab roe. Table S1: The non-polar and semi-volatile metabolites profiles of the freeze-dried blue crab roe, sea urchin roe and beluga caviar.
