# Supplementary material for: Freeze-Drying Blue Crab Roe, Sea Urchin, and Beluga Caviar: Impact on Nutritional, Biochemical, and Sensory Properties
Source: Mar Drugs. 2026 Apr 12;24(4):135. doi: 10.3390/md24040135 (PMC13117789; doi:10.3390/md24040135)
Supplement: Supplementary file 1 [file marinedrugs-24-00135-s001.zip › marinedrugs-4240758-supplementary.pdf]

## Supplementary Material

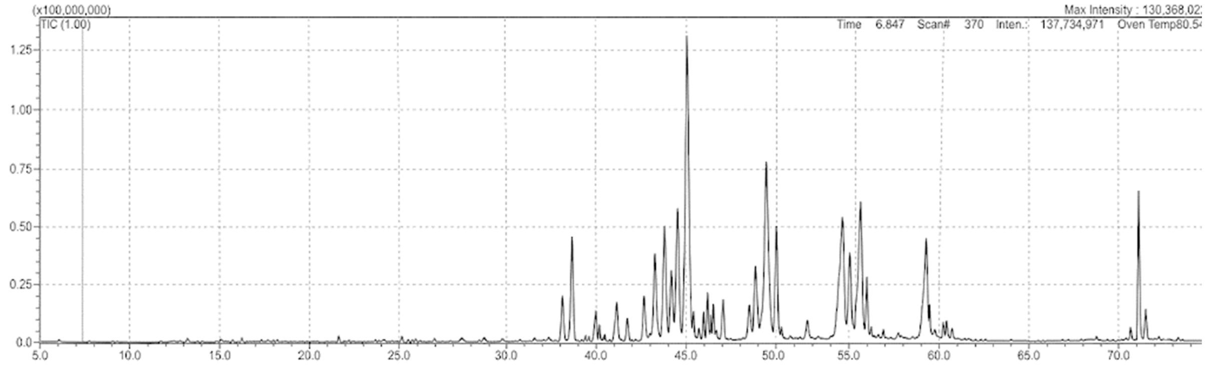


**Figure S1.** Representative Total Ion Chromatograms (TIC) of the volatile compounds identified in the freeze-dried sea urchin.

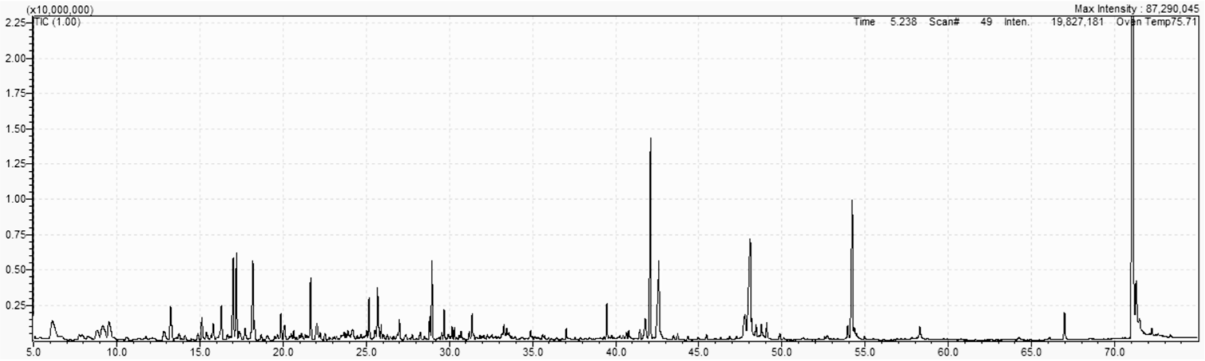


**Figure S2.** Representative Total Ion Chromatograms (TIC) of the volatile compounds identified in the freeze-dried beluga caviar.

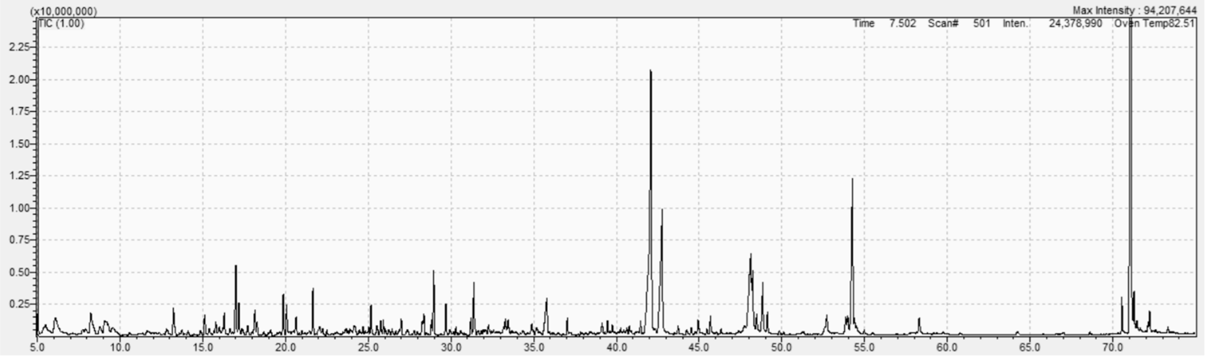


**Figure S3.** Representative Total Ion Chromatograms (TIC) of the volatile compounds identified in the freeze-dried blue crab roe.

**Table S1**. The non-polar and semi-volatile metabolites profiles of the freeze-dried blue crab roe, sea urchin roe and beluga caviar.

| | Compounds | FD Blue crab roe (%) | FD Sea urchin roe (%) | FD Beluga caviar (%) |
|---|---|---|---|---|
| | **Aldehydes** | | | |
| **1** | 7-Tetradecenal, (Z)- | ND | 67.56 ± 0.90 | ND |
| **2** | cis,cis-7,10-Hexadecadienal | ND | 25.93 ± 0.18 | ND |
| **3** | 2,4-Decadienal, (E,Z)- | 6.34 ± 0.08 | ND | ND |
| **4** | 2,4-Decadienal, (E,E)- | 5.90 ± 0.06 | ND | ND |
| **5** | (E,Z,Z)-2,4,7-Tridecatrienal | 2.82 ± 0.02[a] | ND | 4.55 ± 0.22[b] |
| **6** | 2-Octenal, (E)- (trans-2-Octenal) | 2.52 ± 0.01 | ND | ND |
| **7** | cis,cis,cis-7,10,13-Hexadecatrienal | ND | ND | 2.17 ± 0.08 |
| **8** | trans,trans-2,4-Dodecadienal | ND | ND | 1.95 ± 0.01 |
| **9** | NONENAL | 1.41 ± 0.08 | ND | ND |
| **10** | 2,3,7-Trimethyloctanal | ND | 0.63 ± 0.01 | ND |
| **11** | Pentadecanal | ND | ND | 0.38 ± 0.00 |
| | **Ketones, Lactones, Esters & Nitrogen Compounds** | | | |
| **1** | 3,5-Octadien-2-one, (E,E)- | 10.39 ± 0.14 | ND | ND |
| **2** | Perilla acetate | ND | ND | 6.06 ± 0.90 |
| **3** | Limonen-10-yl acetate | 0.92 ± 0.08[a] | ND | 1.66 ± 0.10[b] |
| **4** | trans-.beta.-Ionone | 1.61 ± 0.00 | ND | ND |
| **5** | 2(4H)-Benzofuranone derivative | 1.10 ± 0.02 | ND | ND |
| **6** | Cyclododecanone, 2-methylene- | ND | ND | 1.07 ± 0.04 |
| **7** | Jasmone | 0.96 ± 0.08 | ND | ND |

| | | | | |
|---|---|---|---|---|
| **8** | 3-Buten-2-one, 4-(2,2,6-trimethyl-7-oxabicyclo[4.1.0]hept-1-yl)- | ND | ND | 0.66 ± 0.00 |
| **9** | Cyclohexanone, 2-methyl- | 0.41 ± 0.00 | ND | ND |
| **10** | Benzaldehyde methylimine | ND | 0.30 ± 0.90 | ND |
| **11** | 2-Piperidinone (CAS) 2-Piperidone | ND | 0.20 ± 0.90 | ND |
| **12** | 10-Undecenoic acid, methyl ester | ND | ND | 0.19 |
| **13** | Dihydroactinidiolide | ND | 0.08 ± 0.00 | ND |
| **14** | 3-Methylene-1-oxa-spiro[4.5]decan-2-one | 0.08 ± 0.01 | ND | ND |
| **15** | 4H-Pyran-4-one derivative | ND | 0.04 ± 0.00 | ND |
| **16** | 2-Pyrrolidinone (CAS) Pyrrolidone | ND | 0.03 ± 0.00 | ND |
| | **Alcohols & Acids** | | | |
| **1** | 2-Octen-1-ol, (Z)- (cis-OCT-2-ENOL) | 2.48 ± 0.12 | ND | ND |
| **2** | 1-Octanol, 2,2-dimethyl- | 0.21 ± 0.02[a] | ND | 2.32 ± 0.18[b] |
| **3** | Nepetadiol | ND | ND | 1.71 ± 0.09 |
| **4** | 1-Octanol, 2,7-dimethyl- | 0.21 ± 0.02 | ND | ND |
| **5** | 9-Octadecen-1-ol, (Z)- | ND | ND | 1.25 ± 0.04 |
| **6** | 1-Octanol, 2-butyl- | 1.11 ± 0.00[a] | 0.37 ± 0.01[b] | ND |
| **7** | 2-Ethyl-1-dodecanol | ND | ND | 0.91 ± 0.02 |
| **8** | 1-Dodecanol, 3,7,11-trimethyl- | 0.74 ± 0.01[a] | ND | 0.84 ± 0.04[b] |
| **9** | 9,12,15-Octadecatrien-1-ol, (Z,Z,Z)- | ND | ND | 0.71 ± 0.001 |
| **10** | 1-Heptanol, 2,4-diethyl- | ND | ND | 0.69 ± 0.001 |
| **11** | 1-Octanol, 3,7-dimethyl- | ND | ND | 0.59 ± 0.00 |
| **12** | 11-Methyldodecanol | ND | 0.18 ± 0.01[a] | 0.51 ± 0.01[b] |
| **13** | Methyl salicylate | ND | 0.48 ± 0.02 | ND |

| | | | | |
|---|---|---|---|---|
| **14** | 8-Hydroxytricyclo[5.2.1.0(2,6)]decane | ND | ND | 0.46 ± 0.02 |
| **15** | 11-Tetradecen-1-ol, (E)- | ND | ND | 0.41 ± 0.01 |
| **16** | 1-Decanol, 2-hexyl- | 0.39 ± 0.01 | ND | ND |
| **17** | n-Decanoic acid | ND | 0.30 ± 0.01 | ND |
| **18** | 6-Undecanol | 0.22 ± 0.00 | ND | ND |
| **19** | Caprylic acid | ND | 0.22 ± 0.00 | ND |
| **20** | 1-Dodecanol, 2-hexyl- | ND | 0.20 ± 0.02 | ND |
| **21** | 1-Dodecanol | ND | 0.17 ± 0.01 | ND |
| **22** | E-11,13-Tetradecadien-1-ol | ND | 0.06 ± 0.00 | ND |
| **23** | 1-Heptanol, 2-propyl- | ND | 0.03 ± 0.00 | ND |
| **24** | Artemisia alcohol | 0.03 ± 0.00 | ND | ND |
| **25** | 1-Tridecanol | ND | 0.02 ± 0.00 | ND |
| **26** | Acetic acid, phenylmethyl ester | ND | 0.02 ± 0.00 | ND |
| | **Hydrocarbons, Phenolics & Others** | | | |
| **1** | Cyclohexane, 1,5-diethenyl-3-methyl-2-methylene- | ND | ND | 15.56 ± 0.24 |
| **2** | 7-Methylene-9-oxabicyclo[6.1.0]non-2-ene | 14.94 ± 0.21 | ND | ND |
| **3** | Phenol, 2,6-bis(1,1-dimethylethyl)-4-methyl- | ND | ND | 12.03 ± 0.14 |
| **4** | Dodecane (n-Dodecane)* | 6.95 ± 0.10[a] | 0.08 ± 0.00[b] | 10.88 ± 0.14[c] |
| **5** | 2-Dodecyne | 6.44 ± 0.12 | ND | ND |
| **6** | Hexadecane, 2,6,11,15-tetramethyl | ND | ND | 5.18 ± 0.08 |
| **7** | Nonane, 5-(2-methylpropyl)- / 5-(1-methylpropyl)- | 4.04 ± 0.08[a] | 0.10 ± 0.00[b] | ND |
| **8** | 1,4,9-Decatriene, (Z)- | 3.95 ± 0.04[a] | 0.05 ± 0.00[b] | ND |

| | | | | |
|---|---|---|---|---|
| **9** | Pentadecane | ND | 0.01 ± 0.00[a] | 3.72 ± 0.08[b] |
| **10** | Z,Z,Z-4,6,9-Nonadecatriene | ND | ND | 3.20 ± 0.04 |
| **11** | Tetradecane (n-Tetradecane) | 2.99 ± 0.04[a] | 0.17 ± 0.01[b] | ND |
| **12** | Phenol, 2,4-bis(1,1-dimethylethyl)- | ND | ND | 2.72 ± 0.02 |
| **13** | Heptane, 2,2-dimethyl- | ND | ND | 2.56 ± 0.00 |
| **14** | 11,11-Dimethyl-spiro[2,9]dodeca-3,7-dien | ND | ND | 2.25 ± 0.02 |
| **15** | Undecane, 5,6-dimethyl- | 2.19 ± 0.02 | ND | ND |
| **16** | Undecane, 2,5-dimethyl- | ND | 0.20 ± 0.00[a] | 2.01 ± 0.04[b] |
| **17** | Dodecane, 4,6-dimethyl- | 1.68 ± 0.02[a] | 0.29 ± 0.02[b] | ND |
| **18** | Octane, 5-ethyl-2-methyl- | 1.66 ± 0.02 | ND | ND |
| **19** | Heptadecane | 1.66 ± 0.01 | ND | ND |
| **20** | Nonadecane | ND | ND | 1.43 ± 0.01 |
| **21** | trans-7-Ethyl-bicyclo[4.3.0]non-3-ene | 0.67 ± 0.01[a] | ND | 1.41 ± 0.02[b] |
| **22** | Undecane, 2,6-dimethyl- | 1.40 ± 0.00 | ND | ND |
| **23** | 1,4,8-Dodecatriene, (E,E,E)- | ND | ND | 1.16 ± 0.01 |
| **24** | Dodecane, 4-methyl- | 1.07 ± 0.02[a] | 0.15 ± 0.01[b] | ND |
| **25** | Nonane, 5-methyl-5-propyl- | 1.05 ± 0.02[a] | 0.05 ± 0.00[b] | 0.38 ± 0.02[c] |
| **26** | Tridecane | 1.05 ± 0.01 | ND | ND |
| **27** | Pentadecane, 3-methyl- | ND | ND | 1.00 ± 0.04 |
| **28** | Nonane, 5-butyl- | 0.95 ± 0.01 | ND | ND |
| **29** | Tetradecane, 4-methyl- | 0.83 ± 0.01[a] | 0.13 ± 0.02[b] | ND |
| **30** | Undecane, 4,8-dimethyl- | 0.80 ± 0.00 | ND | ND |
| **31** | 4,4-Dimethyl octane | ND | ND | 0.78 ± 0.01 |

| | | | | |
|---|---|---|---|---|
| **32** | Pentane, 2,3,4-trimethyl- | ND | ND | 0.74 ± 0.00 |
| **33** | Cyclododecyne | 0.72 ± 0.02 | ND | ND |
| **34** | Decane, 3,8-dimethyl- | 0.70 ± 0.02[a] | 0.09 ± 0.01[b] | 0.15 ± 0.00[c] |
| **35** | Heptane, 2,5,5-trimethyl- | 0.69 ± 0.00 | ND | ND |
| **36** | Undecane, 2,4-dimethyl- | 0.68 ± 0.01[a] | 0.08 ± 0.00[b] | ND |
| **37** | 1-Pentadecene | ND | ND | 0.64 ± 0.02 |
| **38** | 1,E-11,Z-13-Pentadecatriene | ND | ND | 0.57 ± 0.01 |
| **39** | Tetradecane, 5-methyl- | ND | 0.09 ± 0.00[a] | 0.55 ± 0.00[b] |
| **40** | Z,Z,Z-1,4,6,9-Nonadecatetraene | ND | ND | 0.54 ± 0.00 |
| **41** | Undecane, 2,7-dimethyl- | 0.53 ± 0.00 | ND | ND |
| **42** | Decane, 5-propyl- | ND | ND | 0.53 ± 0.00 |
| **43** | Decane, 2,3,5,8-tetramethyl- | ND | 0.04 ± 0.00[a] | 0.52 ± 0.00[b] |
| **44** | 1,5-Cyclooctadiene, 3-(1-methyl-2-propenyl)- | 0.51 ± 0.01 | ND | ND |
| **45** | 2,4-Dimethyldodecane | ND | 0.38 ± 0.00 | ND |
| **46** | Cyclododecane | 0.36 ± 0.02 | ND | ND |
| **47** | 1-Undecene, 7-methyl- | ND | 0.24 ± 0.01 | ND |
| **48** | Dodecane, 2,6,11-trimethyl- | 0.24 ± 0.01[a] | 0.13 ± 0.01[b] | ND |
| **49** | Tridecane, 2-methyl- | 0.15 ± 0.01[a] | 0.21 ± 0.00[b] | ND |
| **50** | Undecane, 3,8-dimethyl- | 0.18 ± 0.00 | ND | ND |
| **51** | Tridecane, 6-methyl- | ND | ND | 0.18 ± 0.01 |
| **52** | Hexadecane | ND | 0.17 ± 0.02 | ND |
| **53** | Trimethyl-2,4,4 hexene-1 | ND | 0.13 ± 0.01 | ND |
| **54** | Decane, 2-methyl- | ND | ND | 0.11 ± 0.00 |

| | | | | |
|---|---|---|---|---|
| **55** | Hexane, 1,1'-oxybis- | ND | ND | 0.11 ± 0.00 |
| **56** | Artemiseole | ND | 0.11 ± 0.00 | ND |
| **57** | Decane, 6-ethyl-2-methyl- | ND | 0.09 ± 0.00 | ND |
| **58** | Tridecane, 5-methyl- | ND | 0.09 ± 0.00 | ND |
| **59** | Pentadecane, 2,6,10-trimethyl- | ND | 0.09 ± 0.00 | ND |
| **60** | Octane, 1,1'-oxybis- | ND | 0.01 ± 0.00 | ND |
